# SOCIOECONOMIC STATUS AND WORKING LIFE EXPECTANCY IN SWEDEN

**DOI:** 10.1093/geroni/igad104.1047

**Published:** 2023-12-21

**Authors:** Robin Högnäs

**Affiliations:** Stress Research Institute, Stockholm, Stockholms Lan, Sweden

## Abstract

Longer life expectancy and fertility decline have increased concerns about the security of old-age pensions. Raising retirement ages is one strategy to offset rising costs, though the option to retire varies considerably by socioeconomic status (SES) and sex. The level of variation in SES may depend the measure used. Also, many workers now transition into retirement slowly, e.g., move from full- to part-time work. Thus, retirement age may not sufficiently capture how long people work. Working life expectancy (WLE)—the expected average number of years worked—better measures total working life. We use data from the Swedish Longitudinal Occupational Survey of Health (SLOSH) from 2008 to 2020 (n=4,940 people age 50+ and n=74,093 person-observations) to examine WLE by SES. We estimate a three-state multistate model (i.e., working, not working, dead) and a four-state model (working part-time, working full-time, not working, and dead); both assume a continuous-time first-order Markov process. Models are sex-stratified models, cross-classified by: 1) occupation; 2) education; 3) income. We find that professionals work full-time 1 year more than routine workers, regardless of sex. The low educated work full-time 1 year less than the highly educated. In our weighted three-state model, where part-time work contributed ½ of full-time work, the difference increased to 1.14 and 1.05 years, respectively. Our unweighted three-state model showed slightly larger education differences. Findings suggest that WLE differs by SES, regardless of sex, and the differences are greater by education than occupation. This has implications for extending working life policies.

